# L-DOPA induces iron accumulation in roots of *Ipomoea aquatica* and *Arabidopsis thaliana* in a pH-dependent manner

**DOI:** 10.1186/s40529-023-00396-7

**Published:** 2023-08-25

**Authors:** En-Jung Hsieh, Siao-Wei Liao, Ching-Yuan Chang, Chu-Han Tseng, Shan-Li Wang, Louis Grillet

**Affiliations:** College of Agriculture and Bioresources, Department of Agricultural Chemistry, National Taiwan University, Building No. 2, Rm. 209A No. 1, Sec. 4, Roosevelt Rd, Taipei, 10617 Taiwan

**Keywords:** L-DOPA, Water spinach, Allelopathy, pH, Iron

## Abstract

**Background:**

Iron deficiency is the leading cause of anemia worldwide, particularly in countries with predominant plant-based diets. Plants constitute the main source of dietary iron. Increasing their iron concentration could reduce the occurrence of anemia. The water spinach *Ipomoea aquatica* is consumed as a vegetable throughout Asia and tolerates high iron concentrations making it an attractive candidate for iron biofortification. L-DOPA is an allelopathic molecule secreted by some legumes. L-DOPA can trigger the expression of Fe deficiency-inducible genes, and could potentially be used as a biostimulant to increase Fe concentration.

**Results:**

L-DOPA significantly affected root growth of water spinach, and triggered a massive accumulation of Fe in roots. Both effects were exacerbated when L-DOPA was dissolved in KOH, which is surprising given that L-DOPA is less stable at high pH. To check whether a higher pH could indeed increase the bioactivity of L-DOPA, we used *Arabidopsis thaliana*, which grows at lower pH than water spinach, and subjected the plants to L-DOPA treatments at pH 5.5 and pH 6.0, which are both within the optimal range for Arabidopsis nutrition. At pH 6.0, the root growth of Arabidopsis was more strongly inhibited than at pH 5.5. We found that at higher pH, L-DOPA oxidizes to form a melanin precipitate.

**Conclusions:**

We concluded that the oxidation of L-DOPA that we observed upon solubilization in KOH, or in nutrient solutions at slightly higher pH produces melanin-related molecules that are more potent than L-DOPA itself to trigger the primary root growth inhibition, Fe uptake and root Fe accumulation in water spinach and Arabidopsis.

## Background

Intensive agricultural systems are causing massive deforestation, water scarcities, biodiversity loss, soil depletion and greenhouse gas emissions (Foley et al. 2011) and cannot deliver sustainable food and agricultural production. The Food and Agriculture Organization of the United Nations (FAO) estimates that 33% of the world’s land is degraded (FAO 2017). Developing eco-friendly practices while maintaining yield is one of the greatest global challenges ever faced by humanity. The use of green manure is a common practice which reduces soil erosion, minimize the use of fertilizers and can even decrease the need for herbicides. Notably, several legumes which can establish symbiosis with nitrogen-fixating rhizobacteria, while also serving as protein-rich animal feed, are popular among farmers. In Europe, the broad bean *Vicia faba* is one of the most widely used cover crop as well as an important livestock feed (Jezierny et al. 2010; Smith et al. 2013). In addition, *V. faba* possess herbicidal properties thanks to the production of an allelopathic molecules, L-3,4-dihydroxyphenylalanine (L-DOPA) which is secreted through the roots into the rhizosphere. L-DOPA acts as a growth inhibitor on a broad spectrum of plant species (Fujii et al. 1991), and it was recently shown to act as a belowground pheromone for anti-herbivory communication (Cascone et al. 2023). Furthermore, L-DOPA has been previously shown to induce the expression of iron (Fe) deficiency-inducible genes (Golisz et al. 2011) and in particular, the most highly induced gene was IRONMAN1/FE UPTAKE-ENHANCING PEPTIDE3 (IMA1/FEP3), which encodes a regulatory peptide that controls iron homeostasis in land plants (Grillet et al. 2018; Hirayama et al. 2018). The underlying molecular mechanism is unknown but the authors suggested that the reactivity of the catechol group of L-DOPA towards Fe was involved. The most straightforward explanation is that L-DOPA interacts with Fe, subsequently preventing its transport and metabolization by the plant, and thereby triggering a Fe deficiency response. L-DOPA has indeed been shown to reduce ferric iron (Fe^3+^) to it ferrous (Fe^2+^) form, and to form L-DOPA_3_-Fe chelates (Billings et al. 2019). These processes have been mainly studied in animal models, in which L-DOPA is the biological precursor of the catecholamines neurotransmitters including dopamine and adrenaline. For this reason, L-DOPA constitute the main clinical treatment against Parkinson's disease, which is characterized by the loss of dopaminergic neurons. Dopamine itself can chelate iron and has been shown to co-localize with Fe in mouse neurons (Ortega et al. 2007). Catechols have a high affinity for Fe and Fe-binding by L-DOPA likely affects the bioavailability of Fe for the plant. Therefore, due to its catechol group, L-DOPA is a rather unstable molecule which can undergo spontaneous oxidation in presence of ROS (Albrecht et al. 1990), metals (Palumbo et al. 1987) or high pH (Nakagawa et al. 2018). In plant growth medium, it completely degrades within a few hours at pH 7.0, and much slower at pH 6.0 in soil-free system. In soil, the degradation is even faster and also pH-dependent (Furubayashi et al. 2007). This lability is a double-edge sword for the use of L-DOPA as a bioherbicide: on one hand, L-DOPA has a low remanence and would not cause long lasting damage to the environment, but on the other hand, L-DOPA bioactivity might be insufficient or not last long enough in certain soil types. Therefore, the pH-dependent oxidation of L-DOPA is an important question to address to enable its use in agriculture.

Water spinach (*Ipomoea aquatica* Forssk.) is a popular vegetable in Asia and particularly in Taiwan. This species grows well, with minimal care under submerged conditions. It is one of the richest sources of carotenoids and chlorophylls (Wills and Rangga 1996) and the leaves contain an abundance of essential amino acids, which led to its recommendation as an ideal dietary protein source from the WHO (WHO 1973). It can form an Fe plaque at its root surface (Tang et al. 2021), similarly to rice. It was also shown to accumulate mercury (Hg), aluminum (Al), Cadmium (Cd), lead (Pb), arsenic (As) and Fe (Göthberg et al. 2004; Hanafiah et al. 2020; Liao et al. 2021) to high concentrations when grown on contaminated soil. Undernourishment for iron (Fe) in humans is the most frequent cause of Fe deficiency-induced anemia (IDA), affecting more than two billion people worldwide (McLean et al. 2009). Plants are the major source of dietary Fe, and therefore, overcoming Fe malnutrition requires strategies to increase the Fe content in crops. The water spinach is a widely consumed vegetable throughout Asia which can tolerate a high Fe content. In the present study, we investigated whether L-DOPA could be used to increase the Fe content of water spinach, and whether it would cause toxicity symptoms. The optimal growth pH for water spinach is 6.5. At this pH, L-DOPA was reported to fully oxidize within hours (Furubayashi et al. 2007; Nakagawa et al. 2018). The model plant species Arabidopsis grows at more acidic pH with an optimum value ranging from 5.5 to 6, at which L-DOPA oxidation is much slower. By comparing the effect of L-DOPA solubilized at different pH, or on plants grown at different pH, we found that the inhibitory properties of L-DOPA are surprisingly stronger in conditions at which it is least stable. L-DOPA oxidation in the growth medium led to the formation of melanin (Palumbo et al. 1987), a black pigment that is ubiquitously found in all living kingdom. Our data suggests that melanin formation is correlated with the allelopathic power of L-DOPA.

## Methods

### Plant materials and growth conditions

Water spinach, also named water spinach (*Ipomoea aquatica* Forssk.) and Arabidopsis (*Arabidopsis thaliana* (L.) Heynh) accession Columbia (Col-0) were used in the research. Water spinach seeds were germinated in wet germination paper towels in dark at 30 °C for 5 days. Seedlings were transferred to black tubs (eight seedlings per tub). Plants were grown hydroponically in ES (Estelle and Sommerville 1987) nutrient solution composed of 5 mM KNO_3_, 2 mM Ca(NO_3_)_2_, 2 mM MgSO_4_, 2.5 mM KH_2_PO_4_, 14 μM MnCl_2_, 70 μM H_3_BO_3_, 1 μM ZnSO_4_, 0.5 μM CuSO_4_, 0.2 μM Na_2_MoO_4_, 0.01 μM CoCl_2_, 50 μM Fe-EDTA, and 4.7 mM MES buffer. The pH was adjusted to 6.5 for water spinach, and either pH 5.5 or pH 6.0 for Arabidopsis. Water spinach were cultivated in a growth chamber at 28 °C (12 h light/12 h dark). Fourteen-day-old water spinach were treated with 250 µM L-DOPA dissolved either in 0.1 N HCl or 1 M KOH for another 14 days. For Arabidopsis, plants were grown in a growth chamber at 22 °C (16 h light/8 h dark), 14-day-old Arabidopsis plants were transferred to nutrient solutions at pH 5.5 or pH 6.0 containing 250 µM L-DOPA and grown for an additional 3 days. Twenty-eight-day-old water spinach and 17-day-old Arabidopsis plant were harvested for further experiments.

### Quantification of Fe and chlorophyll

Water spinach plants were dissected into roots and shoots; then total Fe was determined by the BPDS method described by Pan et al. (2015) with minor modifications. Plant samples were dried in an oven at 55 °C for 3 days. After dry weight (DW) measurement, the samples were mineralized in 225 µL of 65% (v/v) nitric acid (HNO_3_) at 96 °C for 6 h, followed by adding 150 µL of 30% (v/v) H_2_O_2_ at 56 °C for 2 h. Each sample was diluted with 225 µL of ultra pure water. Samples and standards were mixed in an assay solution contained 1 mM BPDS, 0.6 M sodium acetate and 0.48 M hydroxylamine. The concentrations of Fe^2+^-BPDS_3_ complexes were measured at 535 nm wavelength with a TECAN Infinite M200 Pro spectrophotometer plate reader. The Fe concentration was calculated by plotting OD values against a standard curve produced with a range of 0 to 40 µg of FeCl_3_. The chlorophyll concentration was measured according to Lichtenthaler and Hartmut, 1987. Water spinach shoots were ground into power at 4 °C and extracted with 5 mL 80% (v/v) acetone. The extracts were centrifuged at 15,000 rpm for 15 min, and the absorbance of supernatants was measured at 662, 645, 470 and 750 nm and used to calculate the concentration of chlorophyll using the equations determined by Lichtenthaler and Hartmut 1987.

### FCR activity assay

Measurement of the root ferric chelate reductase (FCR) activity was carried out as described in Grillet et al. 2014. Arabidopsis roots exposed to L-DOPA treatment for 3 days were excised, rinsed in ultra pure water, and submerged in 2 mL assay solution (1 mM MES buffer pH 5.5, 300 µM BPDS, 100 μM Fe(III)–EDTA per sample) into a 24-well plate. The plate was incubated in the dark for 1 h with gentle horizontal shaking. The absorbance of the assay solutions was measured at 535 nm using a TECAN Infinite M200 Pro spectrophotometer plate reader. Fe concentrations were calculated using the equation determined from FeSO_4_ standards ranging from 0 to 100 µM concentrations.

### UHPLC-MS analysis

Arabidopsis root and shoot tissues were harvested, flash frozen in liquid nitrogen, and homogenized in 0.1 N HCl. The supernatant was separated by centrifugation at 15,000 rpm for 10 min at 4 °C. A ACQUITY UltraPerformance LC^®^ (UPLC^®^) system (Waters, Milford, MA, USA) coupled to an Orbitrap Elite (Thermo Scientific) mass spectrometer was used for the LC–MS analysis. The chromatographic separation for samples was carried out using an ACQUITY UPLC BEH Phenyl Column, 1.7 µm, 2.1 × 100 mm column (Waters). The column was maintained at a temperature of 40 °C and 5 μL sample were injected per run. The mobile phase A consisted in 2% acetonitrile (ACN)/0.1% formic acid and mobile phase B contained 99.9% ACN and 0.1% (v/v) formic acid. The elution gradient was implemented with a flow rate 0.4 of mL/min and a total analysis time of 6 min with the following parameters: 0.5% B at 0 min, 99.5% B at 4 min, a hold at 99.5% B until 5 min, 0.5% B at 5.01 min, and a hold at 0.5% B until 6 min. General instrumental conditions were sheath gas, auxiliary gas, and sweep gas of 35, 15, and 1 arbitrary unit, respectively; ion transfer tube temperature of 360 °C; vaporizer temperature of 350 °C; and spray voltage of 3200 V in positive mode. For analysis, a full MS scan mode with a scan range m/z from 70 to 1000, resolution 15,000 was applied. The Xcalibur4.1 software (Thermo Scientific) was used for the data processing.

### High-Performance liquid chromatography (HPLC) analysis

The detection of L-DOPA by HPLC was conducted on a Hitachi HPLC D-2000 system (Hitachi High-Technologies Corporation, Tokyo, Japan) which is composed of a L-2455 diode array detector, a L-2200 autosampler, and a L-2130 pump. A reverse phase C_18_ column, Cosmosil 5C_18_-AR-II (4.6 × 250 mm, 5 µm, Waters, Milford, MA, USA), was used at 33 °C. The mobile phase consisted of solvent A (pH 2.0 H_2_O adjusted with phosphoric acid) and solvent B (70% methanol). The injection volume was 10 µL and the wavelength was monitored at 282 nm. The elution gradient was programmed as followed: the flow rate was 1.2 mL per minute, 100% solvent A for 6 min, then 100% solvent B for 5 min.

### Synchrotron-radiation-based Fourrier-transform infrared spectroscopy (SR-FTIR) analysis

Melanin (FM67974) from *Sepia officinalis* was purchased from Biosynth AG (Switzerland). L-DOPA (D9628) was purchased from Sigma-Aldrich (USA). DOPA-melanin was produced from L-DOPA by solubilizing it at pH 8.7 in an erlenmeyer flask and letting it oxidize for 72 h with orbital shaking at room temperature. The resulting thick and dark brown solution was then dried using a vacuum concentrator. The analysis was performed at the TLS14A beamline of the National Synchrotron Radiation Research Center (NSRRC, Hsinchu, Taiwan). One milligram of analyte was incorporated into 150 mg of potassium bromide (KBr) with a mortar and pestle, and then compressed into a tablet which was analyzed using a Nicolet 6700 spectrometer (ThermoScientific, USA). The spectra were displayed using OMNIC™.

## Results

### L-DOPA is unstable under alkaline condition

**Fig.1:**
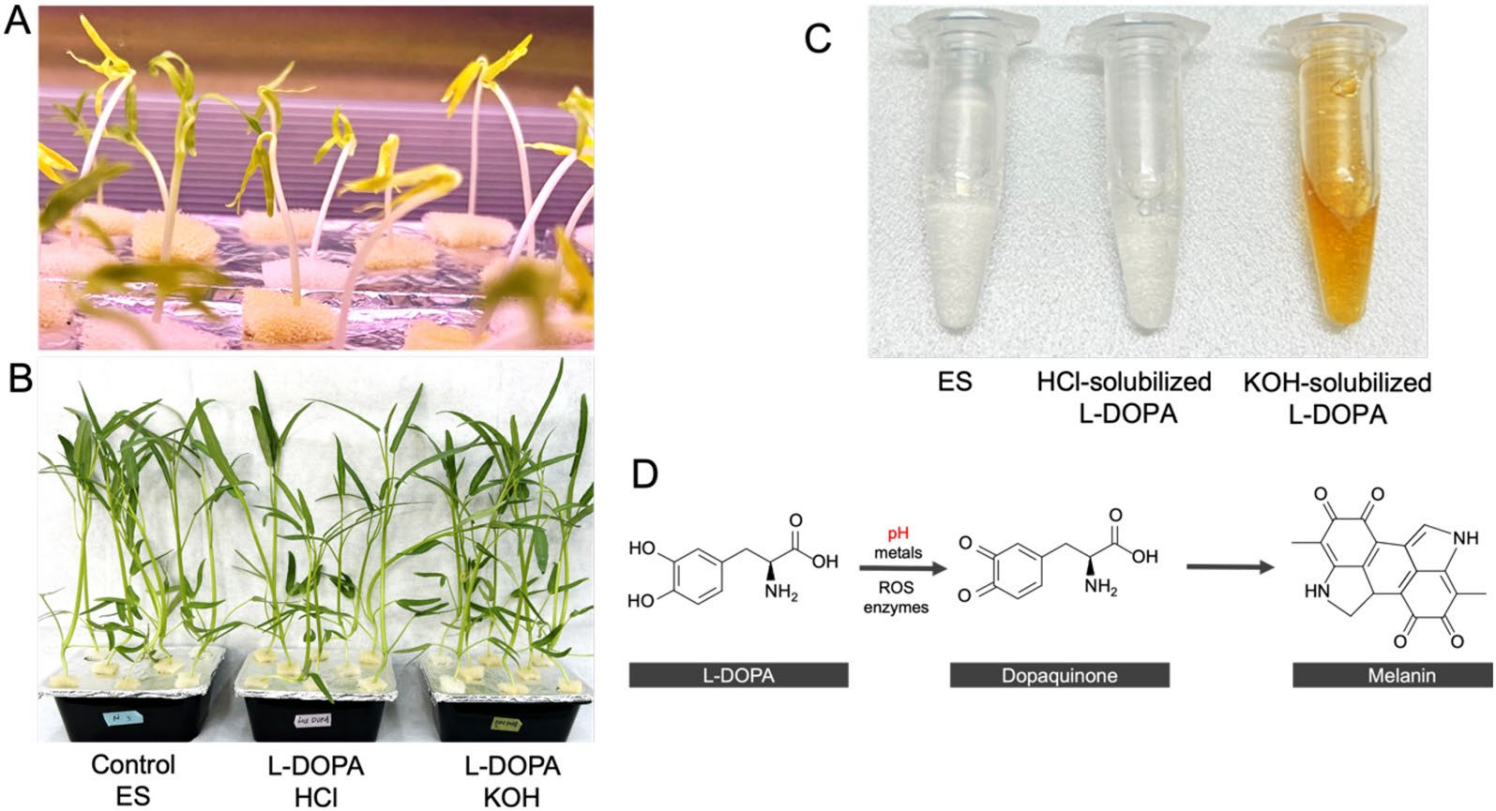
Growth of water spinach (*Ipomoea aquatica* Forssk.) plants subjected to exogenous LDOPA. **A** Seedlings were germinated on sponges and transferred to black boxes containing ES medium. **B** 17 day-old water spinach plants, after 3 days of transfer to L-DOPA containingmedium. **C** Tubes containing the nutrient solution and L-DOPA solubilized either in HCl or in KOH. **D** Overview of the oxidation pathway of L-DOPA

Water spinach (*Ipomoea aquatica* Forssk.) plants were grown in presence of 250 µM L-DOPA. Five-day-old water spinach seedlings were transferred to black containers with ES medium and grown for 9 days (Fig. 1A). Plants were then transferred to ES growth medium containing 250 µM L-DOPA dissolved in either 0.1 N HCl or 1 M KOH and grown for 2 additional weeks (Fig. 1B). We found that L-DOPA dissolved in 0.1 N HCl remained transparent, while the color of L-DOPA in 1 M KOH developed a golden light brown color within minutes (Fig. 1C), indicating a change of chemical state specifically triggered by the alkaline pH. This change was previously described as corresponding to the formation of dopachrome (Palumbo et al. 1987).

### Phenotypes of water spinach grown with L-DOPA

**Fig.2:**
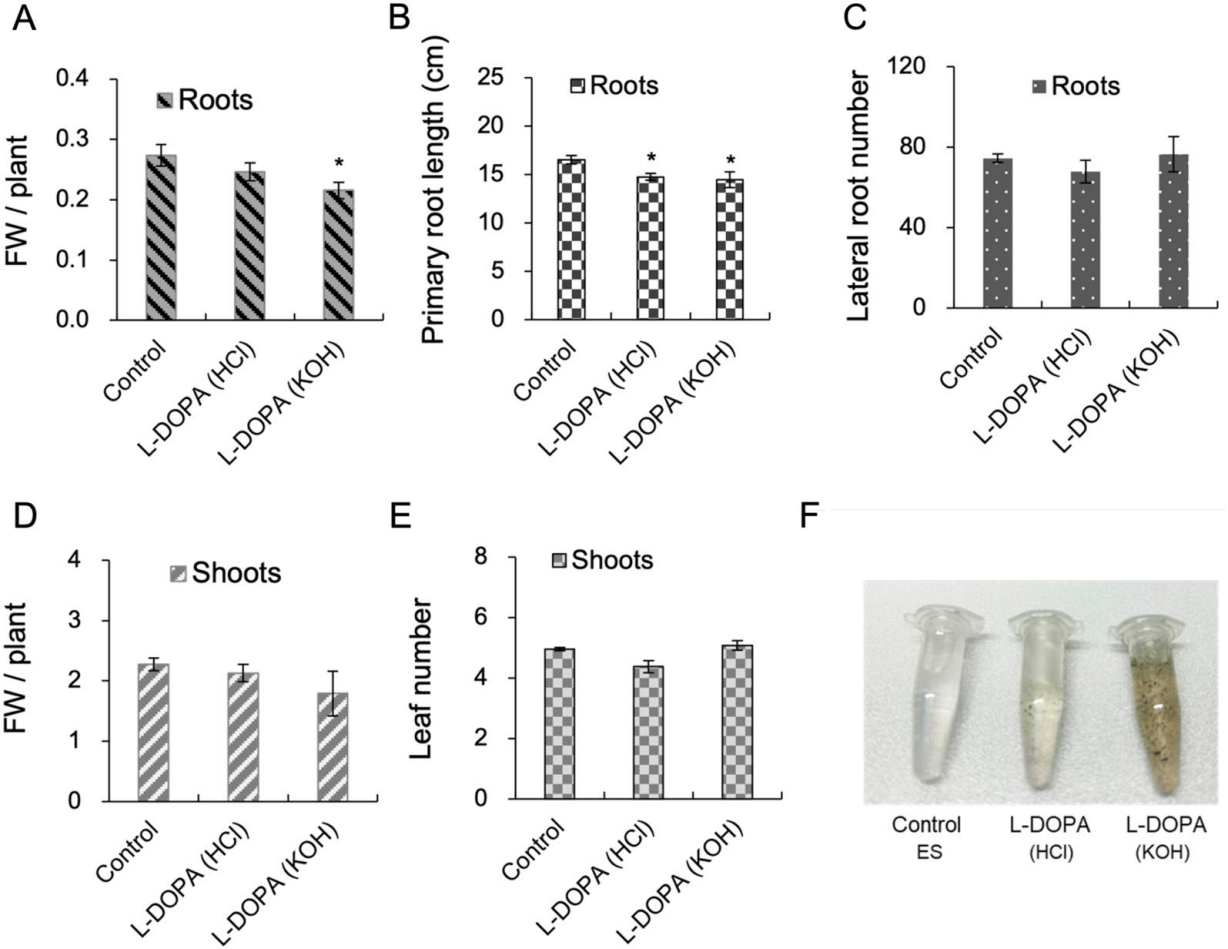
Phenotypes of 28-day old water spinach. **A** Root fresh weight was significantly lower and **B** the length of primary root was significantly shorter in L-DOPA-treated plants. **C** The number of lateral roots, **D** shoot fresh weight and **E** number of leaves were not significantly different between control plants and treated plants. **F** Three tubes containing ES growth medium without treatment (left), and after 4 days of treatment with either L-DOPA dissolved in 0.1 N HCl (middle) or in 1 M KOH (right). The medium containing KOH-dissolved L-DOPA was noticeably darker and contained a black precipitate. Error bars represent the standard deviation (Student’s *t*-test. * P < 0.05, n = 8)

The fresh weight (FW) of both roots and shoots of water spinach were lower in plants subjected to L-DOPA treatment for two weeks, as compared to plants grown on control medium. This effect was more pronounced if L-DOPA was dissolved in KOH (Fig. 2A, D). The primary root lengths of both L-DOPA treatments were shorter than non-treated plants (Fig. 2B). By contrast, the number of leaves and the lateral root formation were not affected by L-DOPA treatment (Fig. 2C, E). The nutrient solution containing L-DOPA dissolved in HCl turned gray, and became darker throughout the following 3 days (Fig. 2F). Medium containing KOH-solubilized L-DOPA was visibly darker and some dark precipitates formed over the course of 3 days. Therefore, the potency of the root growth inhibition seemed to be correlated with the intensity of the black coloration.

### L-DOPA triggers Fe uptake

**Fig.3:**
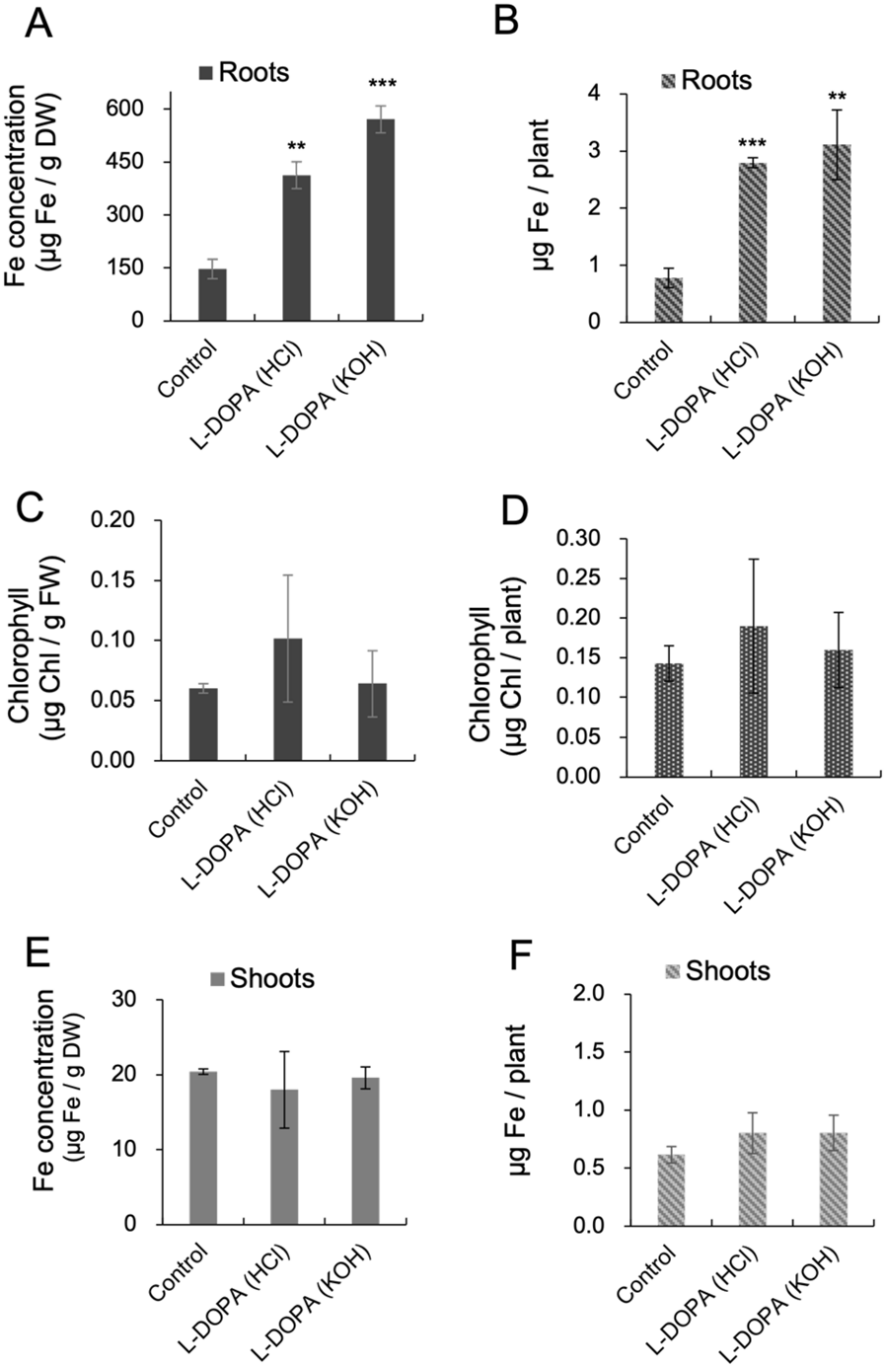
Iron and chlorophyll status in water spinach plants after L-DOPA treatment. **A** Iron concentration in roots. Fe accumulated in roots following L-DOPA treatments, and this effect was stronger with L-DOPA solubilized in KOH. **B** The root Fe content was also affected, showing that the increased Fe concentration resulted from an increased Fe quantity taken up by plants rather than roots being shorter. **C** Chlorophyll concentration in shoots was not affected by L-DOPA. **D** Chlorophyll content was also not affected. **E** Iron concentration in shoots and **F** Iron content of shoots per plant were not affected following L-DOPA treatment. Error bars represent the standard deviation (Student’s *t*-test. ** P < 0.01 and *** P < 0.001; n = 8)

L-DOPA treatment caused a large increase of Fe concentration (Fig. 3A) in roots of water spinach, as compared to roots of plants grown under control conditions, suggesting L-DOPA facilitated Fe uptake into roots. The root content (Fig. 3B) was also increased, suggesting that this effect was not due to the roots being smaller, but that the quantity of Fe that was taken up by the plants was indeed higher. Similarly, to the results obtained on root growth, plants subjected to KOH-solubilized L-DOPA had a higher Fe concentration. However, this increase was not observed in the shoot parts (Fig. 3D, E), in which no significant difference of Fe concentration was observed. Therefore, the fresh weight of shoots, leaf number, and chlorophyll content/concentration was not significantly different as compared to untreated plants (Fig. 2D, E; Fig. 3C, F). In conclusion, L-DOPA did not seem to affect the leaves of water spinach, in contrast to what was observed on roots.

### Higher pH with L-DOPA inhibited plant growth

**Fig.4:**
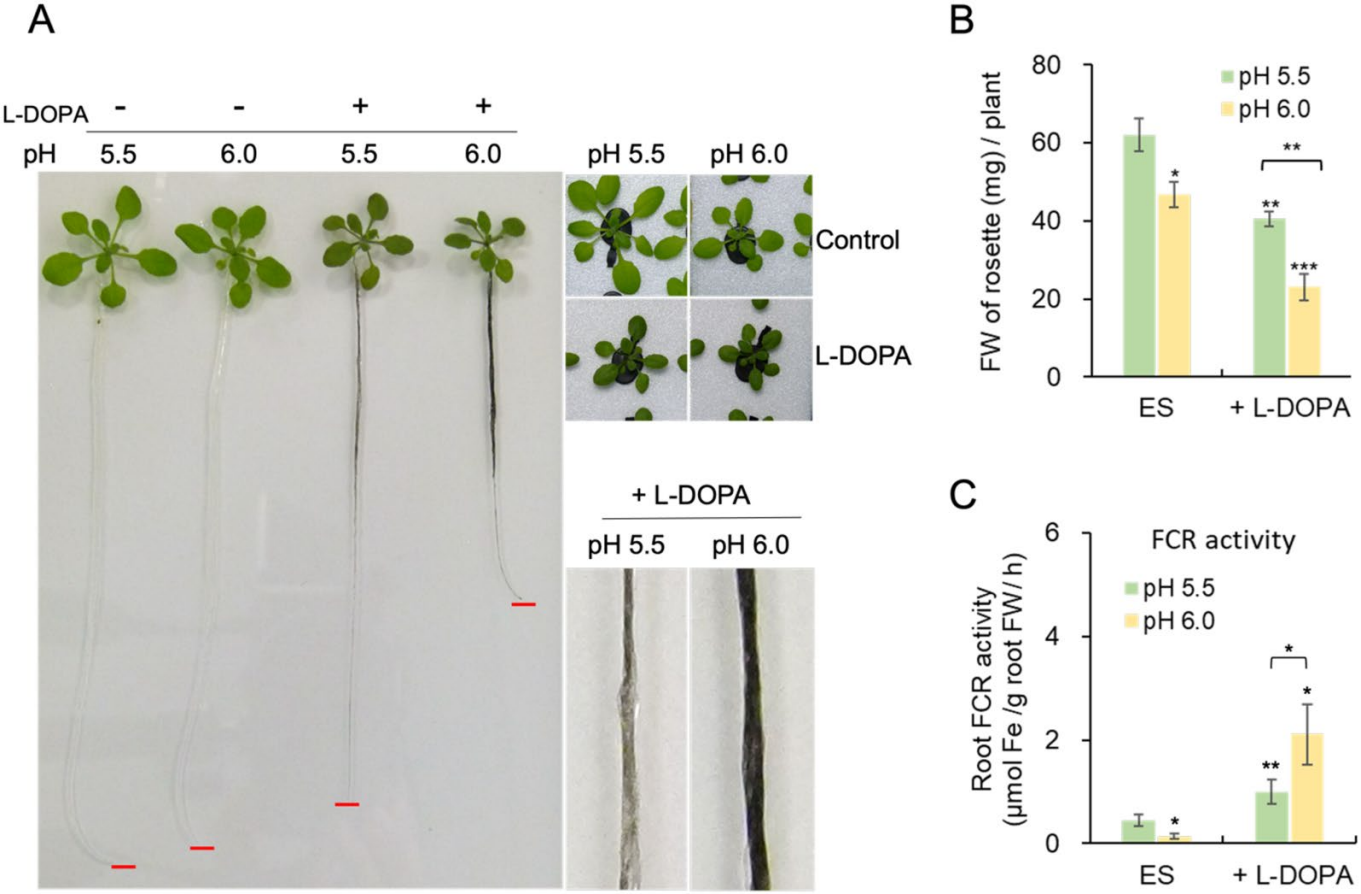
*Arabidopsis thaliana* (Col-0) were grown hydroponically for 14 days and transferred to nutrient solutions with or without L-DOPA, at pH 5.5 and 6.0. **A** Pictures of the plants three days after transfer to the different treatments in a representative experiment. DOPA-treated plants appear smaller and darker than control plants that grew without L-DOPA at both pH. The roots and rosettes of plants treated with L-DOPA at pH 6 are smaller than their counterparts from plants treated at pH 5.5. **B** Fresh weight of rosettes showing the inhibitory effect of L-DOPA on plant growth. The inhibition of rosette size by L-DOPA was exacerbated at pH 6.0. **C** Ferric chelate reductase (FCR) activities of Arabidopsis roots, which constitutes an estimate of the Fe uptake activity. L-DOPA increased FCR activity at the root surface, regardless of the pH. At pH 6.0, the effect of L-DOPA on Fe reduction by roots was more pronounced. The symbols *, ** and *** indicate statistical significance of P < 0.05, P < 0.01 and P < 0.001 respectively. Results are means of the results of 3 experiments, and error bars correspond to standard deviation (n = 3)

The increased oxidation of L-DOPA at higher pH is well-documented. To investigate the influence of small pH differences on the potency of L-DOPA, we carried out another experiment on *Arabidopsis thaliana*, which optimally grows at a lower pH than water spinach. L-DOPA was solubilized in HCl, and added into nutrient solutions at pH 5.5 and pH 6.0, which are both in the optimal pH range for Arabidopsis growth, but are significantly different in terms of L-DOPA oxidation. At both pH, the roots of plants subjected to L-DOPA was shorter than the root of non-treated plants. Interestingly, the roots of L-DOPA-treated plants were shorter at pH 6.0 than at pH 5.5 (Fig. 4A), consistent with an increased inhibitory effect of L-DOPA associated with a higher pH. The rosette fresh weight of Arabidopsis was significantly reduced in plants grown in presence of L-DOPA, at both pH. The plants were visibly smaller and darker as well. Furthermore, the growth was more severely affected by L-DOPA at pH 6.0 than at pH 5.5 (Fig. 2D, E; Fig. 4A, B). The color of the roots appeared noticeably darker at pH 6.0 (Fig. 4A). This observation confirms that L-DOPA oxidation was enhanced by pH. The root ferric chelate reductase (FCR) activity is a rate-limiting enzymatic activity that is coupled to iron uptake in non-grass plant species. It is often used as an estimate iron uptake, because iron uptake measurement requires the use of radioactive iron and is therefore more difficult to implement. FCR was significantly two-fold different between control and L-DOPA-treated plants at pH 5.5 (Fig. 4C). Furthermore, at pH 6.0, the FCR activity of treated plants was approximately 15-fold higher than the activity of control plants, comforting the hypothesis that L-DOPA is more potent at higher pH.

### Oxidation of L-DOPA

**Fig.5:**
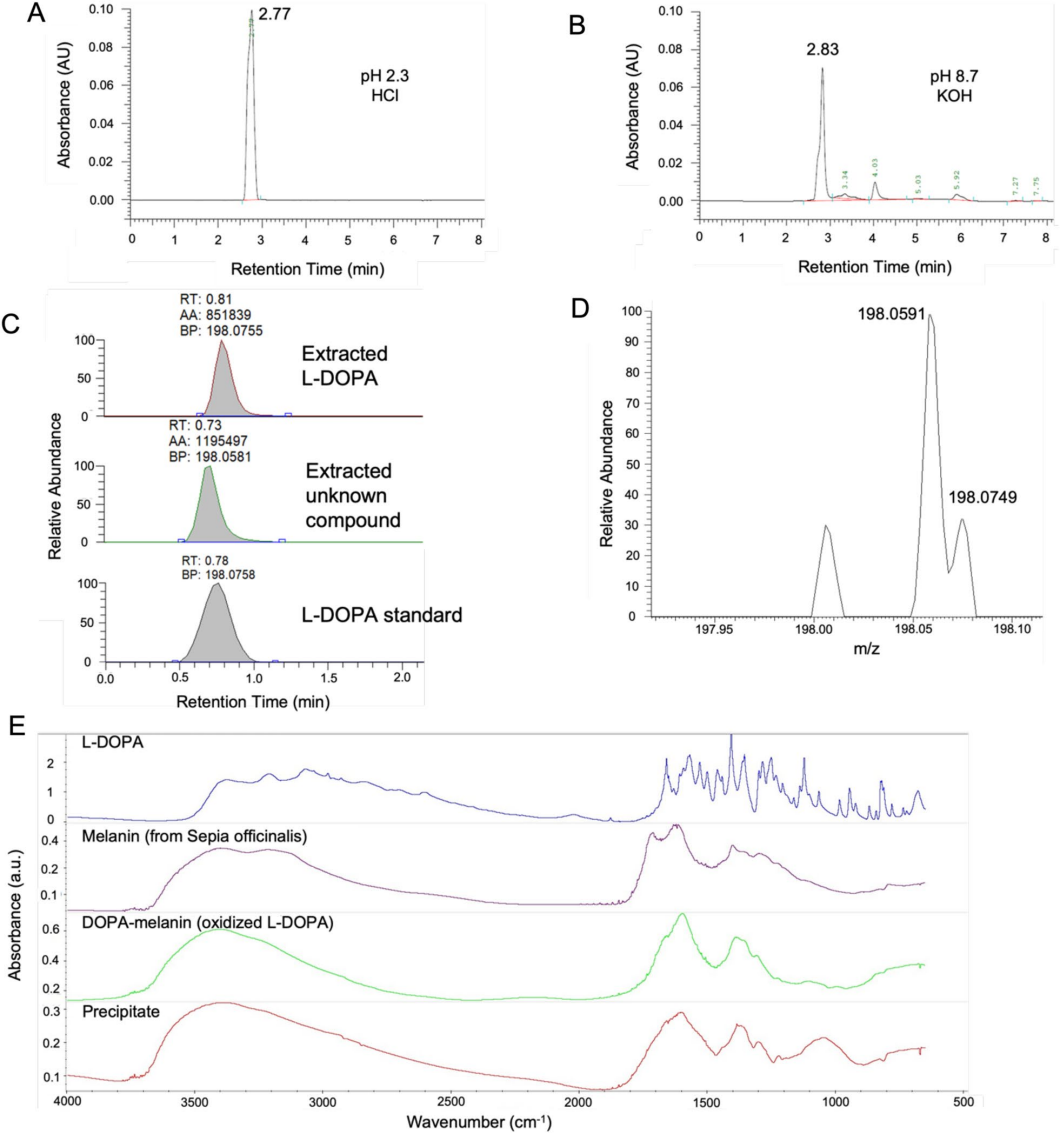
Analysis of L-DOPA and its oxidation products and the influence of pH. **A** HPLC analysis of L-DOPA 10 min after solubilization in HCl at pH 2.3 and **B** in KOH at pH 8.7, corresponding to the two lowest pKa of L-DOPA. The experiment was carried out three times. **C**, **D** UHPLC-MS analysis of L-DOPA extracted from roots of Arabidopsis plants. Two compounds with very close elution time and m/z were detected. **E** SR-FTIR analysis of the dark precipitate forming in nutrient solution containing L-DOPA and of L-DOPA, melanin and DOPA-melanin. The precipitate has absorption properties similar to melanin and DOPA-melanin but distinct from them

To investigate the influence of pH on the oxidation of L-DOPA, we measured L-DOPA concentration using High Pressure Liquid Chromatography (HPLC) after solubilization in either HCl (Fig. 5A) or KOH (Fig. 5B). We found that 10 min after solubilization with KOH, the L-DOPA concentration was lower than after solubilization in HCl, and the elution time of L-DOPA was slightly shifted, suggesting a subtle change of polarity of the molecule. We subsequently extracted L-DOPA from roots of plants grown in nutrient solution containing L-DOPA for 3 days, and attempted to detect L-DOPA using UHPLC-MS. Two molecules producing nearly overlapping peaks were observed (Fig. 5C). L-DOPA standard had a retention time of 0.78 min, while the two other molecules had elution times of 0.81 and 0.73 respectively. This result is consistent with the previous HPLC result, that shows two forms of L-DOPA. The peak detected at 0.81 min likely corresponds to the L-DOPA standard, whereas the molecule eluting at 0.73 might correspond to a transient oxidation intermediate form. The LC–MS spectra of these two peaks revealed that one of them corresponded to an ion with a m/z value of 198.0749, which is almost identical to the predicted L-DOPA mass of 198.0766 m/z. The second peak ion had a m/z of 198.0591 (Fig. 5D) which corresponds approximately the mass of L-DOPA minus three electrons. The mass did not correspond to dopaquinone, dopa semiquinone, or dopachrome, which are the first oxidation products of L-DOPA. L-DOPA is prone to oxidation, which leads to the formation of melanin. The development of a black precipitate in the nutrient solution was correlated with exposure of L-DOPA to higher pH, either during the solubilization or in the nutrient solution, as well as increased root growth inhibition of both Arabidopsis and water spinach. The precipitate was isolated and analyzed using SR-FTIR Spectroscopy, and its absorption spectrum was compared to the spectra of L-DOPA, melanin and DOPA-melanin. The spectra of L-DOPA, melanin and DOPA-melanin were consistent with the spectra reported previously by other groups (Zhou et al. 2013; Bridelli et al. 1999; Turick et al. 2008). The spectrum of the dark precipitate was similar to both spectra of melanin and DOPA-melanin, but very different from the L-DOPA spectrum, suggesting that the later molecule was absent. DOPA-melanin was the closest to the precipitate in terms of infrared absorption properties. The absorption spectrum of the precipitate had however a distinctive broad peak in the 1000–1100 nm region, suggesting a structural difference between L-DOPA solubilized at alkaline pH in vitro and in the nutrient solution.

## Discussion

The effect of pH on the oxidation kinetics of L-DOPA is well documented. The catechol group of L-DOPA has a pKa of 2.39 and upon deprotonation, L-DOPA will form a semiquinone that can subsequently oxidize to a quinone, which has been suggested to cause damages to other molecules and be responsible for the deleterious effects of L-DOPA on plant growth (Soares et al. 2014). In the present study, we observed that the solubilization of L-DOPA in KOH led to a change in the L-DOPA structure, which resulted in the formation of brown compounds and an enhanced bioactivity. The newly formed compound or one of its precursor had a slightly increased retention time in chromatography and was distinct from L-DOPA. The MS analysis showed that the ion had a mass close to the mass of L-DOPA, but with a small difference which was approximately 0.016 Daltons, in the range of 3 electrons. The observed molecule was unlikely to correspond to DOPA semiquinone or dopaquinone, which respectively lost one or two protons, and would therefore have mass differences in the range of 1 or 2 Daltons respectively. Nevertheless, the increased formation of this L-DOPA species, either by solubilization in KOH or by higher pH of the nutrient solution, was associated with an enhanced inhibition of root growth, higher Fe uptake activity in Arabidopsis, and increased accumulation of iron in the roots. This finding was very surprising because the pH-dependent oxidation of L-DOPA in growth medium is well documented, and L-DOPA is much more stable at acidic pH (Furubayashi et al. 2007). It was therefore expected that L-DOPA would be more potent at acidic pH, or after solubilization in HCl. The change of pH from 5.5 to 6.0 is within the optimal pH range for Arabidopsis, and was unlikely to affect the bioavailability of nutrients. For the water spinach, the pH was set at 6.5 in both cases. Therefore, the observed differences of growth must have been caused by a change in the L-DOPA oxidation state rather than an effect of the pH on plant nutrition. The color difference of the roots and nutrient solution caused by the exposure of L-DOPA to higher pH was likely due to the formation of a form of melanin, distinct from both eumelanin and DOPA-melanin. The difference was most notable in the 1000–1100 nm wavelength, which is the region of absorption of C-O stretches. It is therefore possible that the difference of absorption in this zone was related to the oxidation state of the catechol group in the final melanin polymers. Melanin is a well-established oxidation product of L-DOPA, and therefore its formation is a clear indicator of the increased oxidation of L-DOPA. It is plausible that the enhanced oxidation of L-DOPA in alkaline environment would lead to the formation of more quinones, such as dopaquinone and dopa semiquinone. The L-DOPA toxicity has previously been attributed to the formation of these quinones, subsequently causing oxidative stress and ultimately reduced growth (Soares et al. 2014). L-DOPA spontaneous oxidation into melanin and its pH dependence is well-established. However, enzymatic activities from plants roots are likely to also contribute to the melanin formation that is observed at more acidic pH, although this appears to be a minor effect as compared to the pH effect.

Exposure to L-DOPA can cause a fast and robust increase in the expression of Fe deficiency-induced genes in Arabidopsis (Golisz et al. 2011). In water spinach, the Fe concentration increased by 3 to fourfold in the roots. This accumulation could mean either that the Fe uptake by roots increased, causing the concentration to raise, or that the transport of Fe into and throughout the plant was affected, leading to a Fe deficiency response from the plant. The Fe concentration in leaves was not significantly different between plant subjected to L-DOPA or control plants, suggesting that the root-to-shoot transport through the xylem was not affected by the treatment. Furthermore, L-DOPA-treated water spinach did not show any symptom of Fe deficiency such as chlorosis, and in fact, the chlorophyll concentration was not significantly affected. If the plants were Fe deficient for one week, they would have a measurable and visible decrease in chlorophyll concentration. The hypothesis that L-DOPA increases Fe uptake is therefore the most plausible. This assumption is consistent with the result obtained in Arabidopsis, in which a measurable increase in FCR occurred after L-DOPA treatment. FCR is the rate-limiting activity for Fe uptake, and an increased activity is synonymous of increased uptake. In conclusion, L-DOPA exposure triggers a Fe deficiency response which leads to an increased Fe uptake and accumulation of Fe in roots. However, L-DOPA does not prevent Fe from reaching the shoots. Another possibility could lie in the inhibition of the transport of Fe into or throughout the phloem. Indeed, a decrease in phloem Fe, such as the one observed by Zhai et al. (2014), in plants with an impaired expression of the OLIGOPEPTIDE TRANSPORTER 3 (OPT3) gene, can cause a constitutive increase of Fe uptake by roots, regardless of the leaves Fe status. L-DOPA could also activate Fe deficiency through a yet unidentified pathway. The mechanism by which L-DOPA triggers the expression of Fe uptake genes remains elusive to date.

Interestingly, most plants do not produce the most ubiquitous form of melanin, which is the eumelanin, a polymer of predominantly indole monomers. They instead produce allomelanin, which is formed from various possible phenolic precursors including catechol, protocatechuic acid and caffeic acid (Gagloleva et al. 2020). The effect of these compounds on Fe uptake is therefore an interesting direction for future work. The precursors for both forms of melanin possess catechol functional groups, and the resulting polymers have a high affinity for metal cations. However, our study suggests that precursors, monomers or oligomers might also possess a direct or indirect signaling function that can activate Fe uptake.

## Conclusions

L-DOPA can promote iron accumulation in roots of water spinach. The concentration of iron in leaves of water spinach was not affected by the treatment, and the leaves did not exhibit any symptom of Fe deficiency. We therefore concluded that the root-to-shoot translocation of Fe was not impaired. L-DOPA does not appear suitable to increase Fe content in the edible parts of water spinach, however it might have a high potential to increase Fe content in species with edible root parts. L-DOPA inhibited root growth of water spinach and Arabidopsis, and triggered Fe uptake by Arabidopsis roots as well. Both the inhibitory effect and the increased Fe uptake by roots were exacerbated if L-DOPA was exposed to alkaline pH, causing its polymerization into melanin. We conclude that L-DOPA oxidation products are likely more potent than L-DOPA itself to increase Fe uptake and inhibit root growth.

## Data Availability

All the data and material are available from the corresponding author upon reasonable request.

## Abbreviations

L-DOPA: L-3,4-dihydroxyphenylalanine
FCR: Ferric-chelate reductase
FW: Fresh weight
DW: Dry weight
HPLC: High performance liquid chromatography
UHPLC-MS: Ultra-high performance liquid chromatography/mass spectrometry
BPDS: Bathophenanthroline disulfonate
